# Cryopreservation of Human iPS Cell Aggregates in a DMSO-Free Solution—An Optimization and Comparative Study

**DOI:** 10.3389/fbioe.2020.00001

**Published:** 2020-01-22

**Authors:** Rui Li, Kathlyn Hornberger, James R. Dutton, Allison Hubel

**Affiliations:** ^1^Department of Biomedical Engineering, University of Minnesota, Minneapolis, MN, United States; ^2^Stem Cell Institute, University of Minnesota, Minneapolis, MN, United States; ^3^Department of Genetics, Cell Biology and Development, University of Minnesota, Minneapolis, MN, United States; ^4^Department of Mechanical Engineering, University of Minnesota, Minneapolis, MN, United States

**Keywords:** algorithms, biomedical engineering, cryopreservation, human induced pluripotent stem cells, Raman spectroscopy

## Abstract

Human induced pluripotent stem cells (hiPSCs) are an important cell source for regenerative medicine products. Effective methods of preservation are critical to their clinical and commercial applications. The use of a dimethyl sulfoxide (DMSO)-free solution containing all non-toxic molecules offers an effective alternative to the conventional DMSO and alleviates pain points associated with the use of DMSO in the cryopreservation of hiPSCs. Both hiPSCs and cells differentiated from them are commonly multicellular systems, which are more sensitive to stresses of freezing and thawing than single cells. In this investigation, low-temperature Raman spectroscopy visualized freezing behaviors of hiPSC aggregates in different solutions. These aggregates exhibited sensitivity to undercooling in DMSO-containing solutions. We demonstrated the ability to replace DMSO with non-toxic molecules, improve post-thaw cell survival, and reduce sensitivity to undercooling. An accelerated optimization process capitalized on the positive synergy among multiple DMSO-free molecules, which acted in concert to influence ice formation and protect cells during freezing and thawing. A differential evolution algorithm was used to optimize the multi-variable, DMSO-free preservation protocol in 8 experiments. hiPSC aggregates frozen in the optimized solution did not exhibit the same sensitivity to undercooling as those frozen in non-optimized solutions or DMSO, indicating superior adaptability of the optimized solution to different freezing modalities and unplanned deviations. This investigation shows the importance of optimization, explains the mechanisms and advantages of a DMSO-free solution, and enables not only improved cryopreservation of hiPSCs but potentially other cell types for translational regenerative medicine.

## Introduction

Human induced pluripotent stem cells (hiPSCs) are an important cell source for cell therapy and regenerative medicine (Jiang et al., 2014; Singh et al., 2015). With the potential to differentiate into all cell types, high-efficiency banking of hiPSCs is critical for downstream clinical and commercial production of cells and tissues for human therapeutic applications. Effective, consistent methods of hiPSC cryopreservation reduce cost and time associated with production of regenerative medicine and enable development of a manufacturing paradigm for these products.

Existing methods of preservation of hiPSCs are not suitable for current and emerging applications. Specifically, conventional methods use dimethyl sulfoxide (DMSO) as a cryoprotective agent (CPA) (Claassen et al., 2009; Baharvand et al., 2010; Li et al., 2018). DMSO increases the mRNA level of the *de novo* DNA methyltransferase DNMT3A in mouse embryoid bodies, accompanied by hyper- or hypo-methylation of many genetic loci (Iwatani et al., 2006) making it unsuitable for use with reprogrammed cells. Furthermore, DMSO is cytotoxic, and it is common practice for cells to be frozen immediately upon introduction of DMSO-containing CPA solutions. After thawing, the cells are typically washed or diluted quickly as well to minimize their exposure to DMSO. These are pain points associated with the use of DMSO in hiPSC-based cell production processes regardless of the type of application.

DMSO-free CPAs have the potential to alleviate the aforementioned pain points and improve the utility, effectiveness and efficiency of hiPSC cryopreservation. We have demonstrated in previous studies that combinations of sugar, sugar alcohols and amino acids can be used to preserve human mesenchymal stem cells and T lymphocytes (Pollock et al., 2016, 2017; Pi et al., 2018, 2019b). When used to cryopreserve cells *in vitro*, they have been shown to act in concert with each other and amino acids to protect and stabilize cells (Pollock et al., 2016; Pi et al., 2019b). This study uses a cocktail of DMSO-free CPAs including sucrose, glycerol, isoleucine, human serum albumin, and poloxamer 188 (P188), which are non-toxic and FDA-approved for infusion.

In this manuscript, the freezing response of hiPSC aggregates preserved in varying DMSO-free CPA solutions is explored. A DMSO-free CPA formulation is optimized using a differential evolution (DE) algorithm. Low-temperature Raman spectroscopy and differential scanning calorimetry (DSC) are used to characterize the freezing behaviors of the hiPSC aggregates as well as the CPA solutions. The optimized DMSO-free method is compared to similar yet non-optimized alternative solutions and benchmarked against a DMSO-based solution under varying freezing conditions.

## Materials and Methods

### Cell Culture

The hiPSC line UMN PCBC16iPS was used in this investigation (Ye et al., 2013). The cells were cultured as adherent colonies on recombinant human vitronectin (Peprotech) in TeSR-E8 medium (STEMCELL Technologies) and passaged every 4 days as multicellular aggregates using enzyme-free dissociation reagent ReLeSR (STEMCELL Technologies). Passage numbers of the cells used were between 59 and 71. Cultures were routinely tested for mycoplasma using the MycoAlert PLUS detection kit (Lonza). G-banding at a 400-band resolution was performed on the cells from both the earliest and latest passages. Cell culture of the latest passage used for karyotyping arose from cells previously cryopreserved and was the result of three freeze-thaw cycles with three passages in between via the optimized DMSO-free cryopreservation method described in this investigation.

### Freezing Solutions

Components of the DMSO-free freezing solutions in this investigation included sucrose (Sigma-Aldrich), glycerol (Humco), L-isoleucine (Sigma-Aldrich), human serum albumin (Albutein, Grifols), poloxamer 188 (P188, Spectrum Chemical), MEM non-essential amino acids (NEAA, Sigma-Aldrich) and Hank's Balanced Saline Solution with Ca^2+^, Mg^2+^, and glucose (HBSS, Lonza). The final working concentrations of the DMSO-free CPA molecules—sucrose, glycerol, isoleucine, and albumin—were varied to optimize the composition of the freezing solution. A basal buffer composed of P188 at a non-micelle forming concentration, NEAA and HBSS were kept constant for all DMSO-free freezing solutions studied. DMSO (Sigma-Aldrich) was used as a control at an optimized concentration of 7.5%, and HBSS was used as its basal buffer. The DMSO-free compositions discussed in this article are covered by issued Patent #10,314,302 and patent application #US2019/0269124, owned by Regents of the University of Minnesota. The solutions used in this article can be acquired directly from the authors for replication of the studies.

### Pre-freeze Cell Processing

The hiPSCs were cultured to a 65–75% confluence on the day of freezing. Freezing studies were performed using small aggregates (3–50 cells). After dissociation, the cell aggregates were collected using the basal buffer. The aggregate size was controlled by the amount of gentle pipetting. The freezing solutions were prepared at twice (2×) the final working concentration and added dropwise to the suspension of aggregates at a 1:1 ratio. The mixture was incubated at room temperature for 30 min to 1 h before freezing to allow sufficient internalization of intracellular CPAs. Working cell concentration for cryopreservation was defined based on surface area of the 2D cell culture as shown in Table 1 (e.g., all cells produced from 1 well of a 6-well cell culture plate were frozen in 1 ml of 1× cryopreservation solution). Exact cell concentration was measured using a hemocytometer (Hausser Scientific) after dissociating a sample of the hiPSC culture into single cells using Gentle Cell Dissociation Reagent (STEMCELL Technologies) and staining the cells using acridine orange and propidium iodide (Invitrogen). The working cell concentration from samples used for cryopreservation in this study was between 1.46 and 1.89 million cells per ml (95% confidence interval).

**Table 1:** Working cell concentration for hiPSC cryopreservation^a^.

**Cultureware**	**Growth surface area**	**Number of cells**	**Step 1. Cell collection**		**Step 2. CPA introduction**		**Number of cryogenic vials**
			**Volume of buffer**	**2 × cell conc**.	**Volume of 2 × CPA solution**	**Final cell conc**.	
6-well plate	9.5 cm^2^	1.5 ×10^6^/well	500 μl/well	3 ×10^6^/ml	500 μl/well	1.5 ×10^6^/ml	1/well
T25 flask	25 cm^2^	4 ×10^6^	1.3 ml		1.3 ml		2
T75 flask	75 cm^2^	1.2 ×10^7^	3.9 ml		3.9 ml		7
T175 flask	175 cm^2^	2.8 ×10^7^	9.2 ml		9.2 ml		18

a *Numerical values in this table are estimates of the respective parameters. CPA, cryoprotective agent*.

### Controlled-Rate Freezing

Cryogenic vials (Nunc CryoTubes, Thermo Scientific) were used to contain cell aggregates suspended in varied freezing solutions for controlled-rate freezing at a final working volume of 1 ml/vial. Vials of cells were frozen using a liquid nitrogen (LN2)-based controlled-rate freezer (Kryo 560-16, Planer) following the steps listed below using a cooling rate, *B*, of −1°C/min and an ice nucleation (or seeding) temperature, *T*_*NUC*_, of −4 (the optimized *T*_*NUC*_ for hiPSCs) or −12°C (a suboptimal *T*_*NUC*_ for comparative studies) (see Supplementary Figure 1 for cooling profile and an automated alternative of this process):

1. Starting temperature 20°C;
−10°C/min to 0°C;
3. Hold at 0°C for 10 min to equilibrate temperature inside and outside vials;
−1°C/min to *T*_*NUC*_;
5. Hold at *T*_*NUC*_ for 15 min to equilibrate temperature inside and outside vials;
6. Induce ice nucleation manually, briefly spraying LN2 onto vials using a Cryogun (Brymill);
7. *B*°C/min to −60°C;
−10°C/min to −100°C.

Sample temperature was logged using the built-in thermocouple of the controlled-rate freezer, which was inserted via a holed, fitted cap into a “dummy” vial containing the same volume of cells and cryopreservation solution as the experimental samples. After freezing, the vials were transferred in a portable LN2 CryoPod Carrier (BioCision) and stored in liquid phase of LN2.

### Passive Freezing

An alternative to controlled-rate freezing, passive freezing was performed using an insulated freezing container CoolCell (BioCision) inside a −80°C mechanical freezer. Vials of cells were placed inside the CoolCell at room temperature and frozen over a period of 4 h. Sample temperature was logged using a DI-245 USB Thermocouple Data Acquisition system (DATAQ). The thermocouple was inserted via a holed, fitted cap into a “dummy” vial containing the same volume of cells and cryopreservation solution as the experimental samples. After freezing, the vials were transferred in the CryoPod Carrier and stored in liquid phase of LN2.

### Thawing and Post-thaw Cell Characterization

Frozen vials were thawed in a 37°C water bath for 2 min 30 s. Thawed cells were immediately diluted dropwise using TeSR-E8 medium, plated onto vitronectin-coated culture vessels without washing and placed into a 37°C 5% CO_2_ incubator. The split ratio at thawing was 1:1 in the DE algorithm experiments and 1:6 otherwise. Twenty-four hours later, the post-thaw culture was washed with PBS containing Ca^2+^ and Mg^2+^ and stained for esterase activity using calcein AM (Invitrogen). Fluorescence intensity of the stained cells were measured using a Synergy HT microplate reader (BioTek) with an ex/em 485/528 nm filter set. Cells cryopreserved using DMSO and plated post-thaw were used as a control. The ratio of fluorescence measurement of each DMSO-free sample to that of the DMSO control (or post-thaw reattachment rate) was used as the metric of optimization. In addition, fresh cells with the same cell count as cryopreserved cells pre-freeze were used as a positive control, passaged at the same split ratio and stained with calcein AM 24 h post-passage.

Growth in post-thaw culture of cells cryopreserved in the optimized DMSO-free solution was assessed label-free by imaging the culture daily with a Cytation 1 cell imaging multi-mode reader (BioTek) with a 4× objective (NA 0.13, Olympus) in the bright-field and scan mode using default focusing method. Images were automatically analyzed by the Gen 5 software (BioTek) using boundary recognition to measure the confluence. On day 4 after thawing as a result of three stress cycles of freezing, thawing and culturing for one passage, the cell colonies were examined for protein expression by immunocytochemistry using antibodies to detect Nanog (R&D Systems) and Oct4 (Millipore), counterstained with Hoechst 33342 (Invitrogen) and quantified using FIJI (ImageJ). Cells dissociated from the post-thaw culture were examined with anti-Tra-1-60 antibody and its isotype control (Invitrogen) and measured using a BD Accuri C6 flow cytometer (BD Biosciences). Also on day 4, the post-thaw culture was differentiated into all three germ layers using a STEMdiff trilineage differentiation kit (STEMCELL Technologies) over 7 days. The endoderm was detected with anti-FOXA2 and anti-SOX17 antibodies (R&D Systems), the mesoderm with anti-BRACHYURY (R&D Systems) and anti-HAND1 (Invitrogen) antibodies, the ectoderm with anti-PAX6 (DSHB) and anti-NESTIN (STEMCELL Technologies) antibodies, and all counterstained with DAPI (Roche). Fluorescent secondary antibodies (Invitrogen) were utilized to detect primary antibody binding and imaged using the Cytation 1 cell imaging multi-mode reader with a 20× objective (NA 0.45, Olympus).

### Algorithm

A DE algorithm with the basic mutation strategy (DE/rand/1/bin) [Storn and Price, 1997; Pollock et al., 2017; Pi et al., 2019a] was used to rapidly optimize the composition of the DMSO-free freezing solution for hiPSCs based on the functional metric of post-thaw reattachment rate. Briefly, the DE algorithm utilizes stochastic direct search to randomly generate an initial group (Generation 0) of sample parameters (i.e., concentrations of DMSO-free CPA molecules) from the population spanning the entire parameter space. Generation 0 samples were tested experimentally, and their post-thaw reattachment rates were used by the algorithm to output the next group (Generation 1) of CPA concentrations that were mutated versions of Generation 0 to be tested. Cumulatively best members of each generation were stored as an emergent population. The algorithm-driven optimization was completed, and convergence was achieved when the emergent population of the latest generation was the same as that of the previous generation. Parameter space of the DE algorithm was determined by the cytotoxicity limit and was discretized into five intervals between 20 and 120 mM for sucrose, 2.5 v./v. and 5% v./v. for glycerol, 0 and 37.5 mM for isoleucine, and 0 and 2.5% for albumin. Cytotoxicity was defined as decrease in cell reattachment in the hiPSC culture measured using calcein AM 24 h after 1-h exposure to the respective molecules compared to fresh cell control.

### Low-Temperature Confocal Raman Microscopy

A Peltier stage (Thermonamic Electronics) and a series 800 temperature controller (Alpha Omega Instruments) were used to freeze samples of hiPSC aggregates in freezing solution or of freezing solution alone for low-temperature confocal Raman microscopy (Li et al., 2018). A cooling rate of −1°C/min was used for cells and −10°C/min for solution alone, and ice nucleation was induced at −4°C by briefly touching a LN2-chilled needle, unless otherwise noted. Final temperature was stabilized at −50°C, where Raman data were collected. Raman spectroscopic measurements were made using the WITec Confocal Raman Microscope Alpha 300R with UHTS spectrometer and DV401 CCD detector with 600/mm grating. A 532 nm Nd:YAG laser was used as the excitation source. A 100× air objective (NA 0.90, Nikon Instruments) was used to focus the laser. Laser power at the objective was measured at 10 mW using an optical power meter (Thorlabs). Spatial resolution of the microscope was measured by WITec (unpublished) at 305 nm in lateral direction and 790 nm axial.

**Figure 1:**
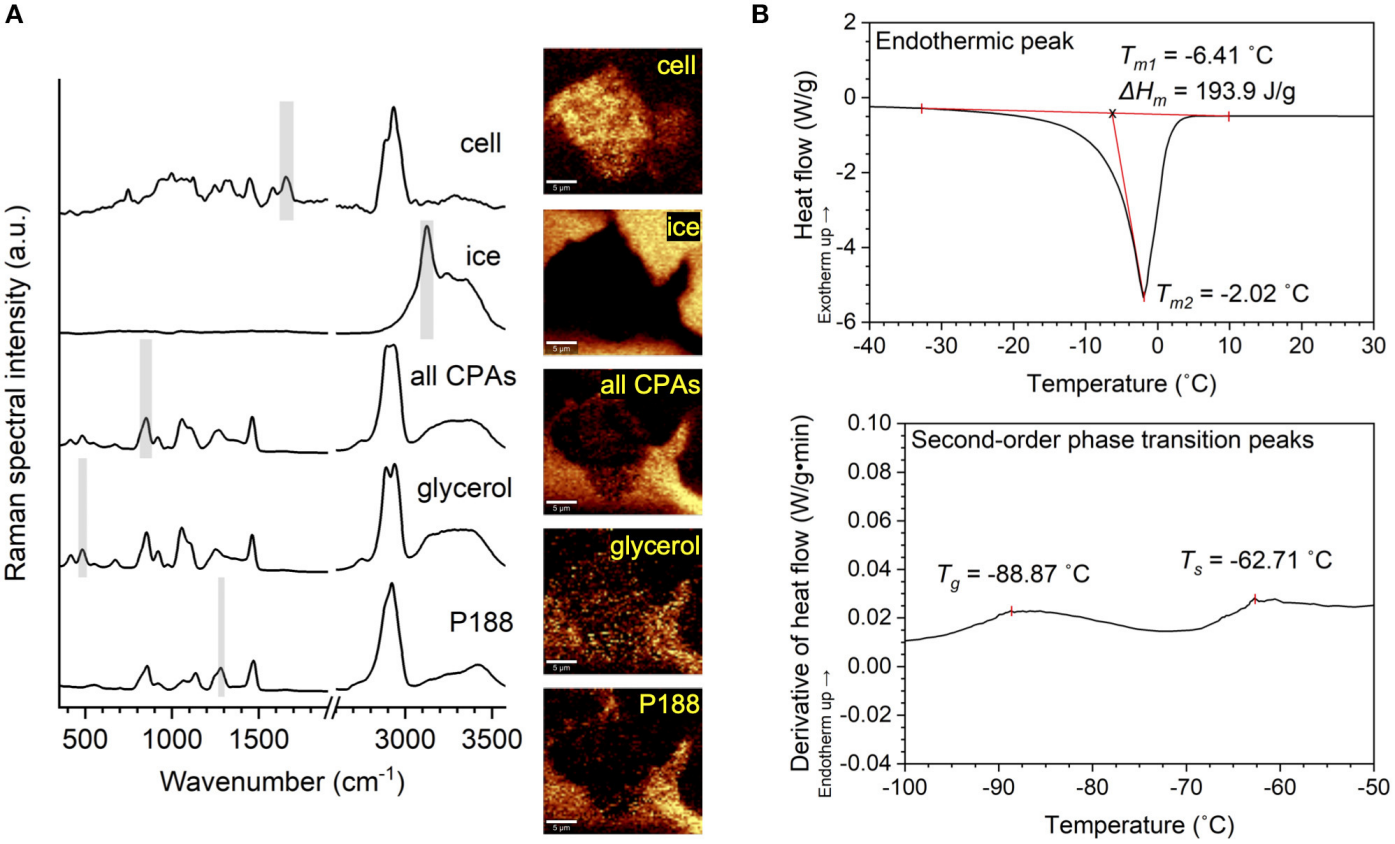
Illustration of Raman and differential scanning calorimetry (DSC) methodology. **(A)** Raman spectra of a hiPSC aggregate, ice, all cryoprotective agents (CPAs) combined, glycerol and poloxamer 188 (P188), as well as Raman heat maps rendered from the spectra based on the characteristic Raman signal shown by shaded region, respectively. **(B)** Top: DSC thermogram showing endothermic peak (melting). The melting temperature was defined as the onset of melting (*T*_*m1*_) for evaluation of thermophysical properties and the peak of melting (*T*_*m2*_) for the interpretation of undercooling. Enthalpy of melting (Δ*H*_*m*_) was defined as the area under the curve of the endothermic peak. Bottom: First derivative of SC thermogram showing second-order phase transitions. Glass transition temperature (*T*_*g*_) and softening temperature (*T*_*s*_) were visualized by deriving heat flow with respect to time and defining *T*_*g*_ and *T*_*s*_ as two local maxima along the derivative curve.

As shown in Figure 1A, Raman heat maps of different substances were rendered by integrating the Raman spectra, acquired at each pixel in the frozen sample, of their characteristic wavenumbers, respectively (Table 2). An integration time of 0.2 s was used to scan each pixel. The size of a pixel was 333 by 333 nm. The rendered Raman heat maps were spatially deconvolved using the theoretical point spread function (PSF) based on the instrumental setup. 2D PSF was generated using the ImageJ macro Diffraction PSF 3D, and spatial deconvolution was performed using the ImageJ macro Iterative Deconvolve 3D. Distribution of the protein and lipid signal was used to delineate the area of the frozen hiPSC aggregate. Raman heat maps of ice were quantitatively analyzed using FIJI, where the ice crystals were identified by thresholding and boundary recognition.

**Table 2:** Raman spectral peak assignments^a^.

**Substance**	**Wavenumber (cm^**−1**^)**	**Assignments (Mathlouthi and Luu, 1980; Mendelovici et al., 2000; Stone et al., 2004; Salzmann et al., 2006; Okotrub and Surovtsev, 2013)**
Proteins, lipids (cell)	1,620–1,700	Amide I and C = C stretching
Ice	3,087–3,162	OH stretching
Glycerol	460–510	CCO rocking
P188	1,267–1,303	CH_2_ twisting
All CPAs (sucrose, glycerol, isoleucine, P188)	815–885	CC stretching
DMSO	648–698	Symmetric CS stretching

a *P188, poloxamer 188; CPA, cryoprotective agent; DMSO, dimethyl sulfoxide; C = C, OH, etc. refer to chemical bonds*.

### Differential Scanning Calorimetry

DSC experiments of CPA solutions were carried out in a Q1000 DSC (TA Instruments). Approximately 5 mg of each sample was encapsulated in an aluminum pan (DSC Consumables) with a hermetically sealed lid. The DSC run for each sample began at 20°C. Samples were cooled to −140°C at a rate of −10°C/min and subsequently heated to 40°C at a rate of +10°C/min. An empty pan was used as a reference. Samples were run in independent triplicates.

TA Universal Analysis Software was used to determine the thermophysical behavior of the CPA solutions. As shown in Figure 1B, enthalpy of melting and melting temperature were calculated from the endothermic melting peak for each sample. Enthalpy of melting was defined as the area under the endothermic peak, and melting temperature was defined as the onset of melting. Glass transition temperature, a second-order phase transition, was determined by plotting the first derivative of heat flow over time against temperature. Two local maxima were typically found of each solution representing the glass transition and softening temperatures (Baboo et al., 2019), respectively in ascending order.

### Statistics

Sample size of 1 was used in the DE algorithm-driven experiments to optimize the DMSO-free CPA formulation with high efficiency. Power analysis was performed for the remaining experiments to ensure sufficient sample size with a power of 0.95. One-sample hypothesis testing was performed on measurements for each sample, where 95% confidence intervals were shown. Error bars represented standard error. Two-tailed Student's *t*-tests were performed for two-sample comparisons, with the exception of a Fisher's exact test performed for two-sample comparisons of samples with binomial distribution. ANOVA with Bonferroni correction was performed for comparisons of multiple samples. Levene's Test was used to verify the assumption for homogeneity of variance for the two or more samples. Null hypothesis was defined as no statistical difference between any pairs of samples or between sample and control. *P*-value < 0.05 was used to make the decision of rejecting the null hypothesis and determining the significant difference.

## Results

### DE Algorithm-Driven Optimization of DMSO-Free CPA Formulation

Optimizing the DMSO-free freezing solution in this study involved using the DE algorithm to identity the composition of CPA variables (sucrose, glycerol, isoleucine, and albumin) associated with the highest post-thaw cell survival. A single cooling rate (−1°C/min) and a single ice nucleation temperature (−4°C) were used in this investigation based on preliminary studies that screened different cooling rates as previously described (Li et al., 2018) with different compositions. The DE algorithm was used with a population size (*NP*, i.e., the number of unique solutions output per generation) of 10, with mutation factor (*F* or weight, i.e., amplification of the differential variance) of 0.85 and crossover (*CR*) of 1. Quantified outcome of a functional assay—post-thaw cell reattachment measured by calcein AM-stained, adherent, live cells in culture 24 h after thawing—was used as the metric of optimization.

**Figure 2:**
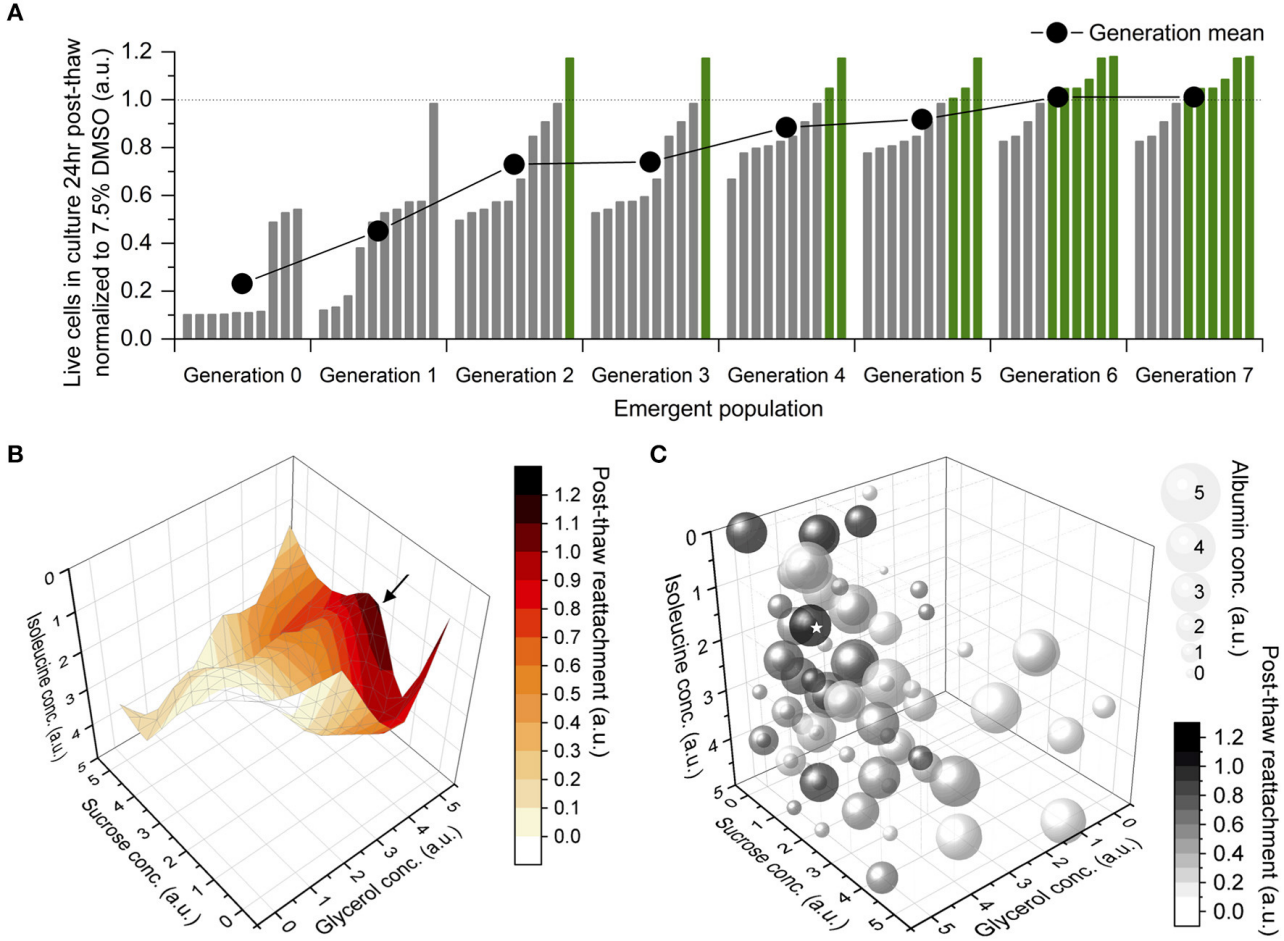
Results of differential evolution (DE) algorithm-driven optimization. Sample size of 1 for accelerated optimization. **(A)** Emergent population of formulations of freezing solution improving then stabilizing in terms of their post-thaw reattachment normalized to a 7.5% dimethyl sulfoxide (DMSO) solution, at the end of which, 6 DMSO-free solutions were better than the DMSO control. **(B)** Topology of parameter space that the DE algorithm navigated through during optimization, shown in four of five dimensions. An optimum (→) was located toward medium concentration of sucrose, high concentration of glycerol and low concentration of isoleucine. **(C)** Bubble chart of all formulations tested by the DE algorithm, shown in all five dimensions. Bubble size correlated to albumin concentration. Grayscale intensity correlated to post-thaw cell reattachment. The optimum (⋆) located at level-2 sucrose, level-5 glycerol, level-1 isoleucine, level-4 albumin.

The mean post-thaw cell reattachment increased with every generation, and the number of DMSO-free freezing solutions better than the 7.5% DMSO control increased gradually, until both plateaued between Generations 6 and 7 (Figure 2A). Therefore, the DE algorithm converged to the global maximum after eight generations, or eight sets of 10-sample experiments. The topology of this parameter space was non-linear and NOT unimodal (Figure 2B). It was complex with closely-spaced contour lines within the region containing the optimum, which was level-2 sucrose, level-5 glycerol, level-1 isoleucine, and level-4 albumin (Figure 2C).

**Figure 3:**
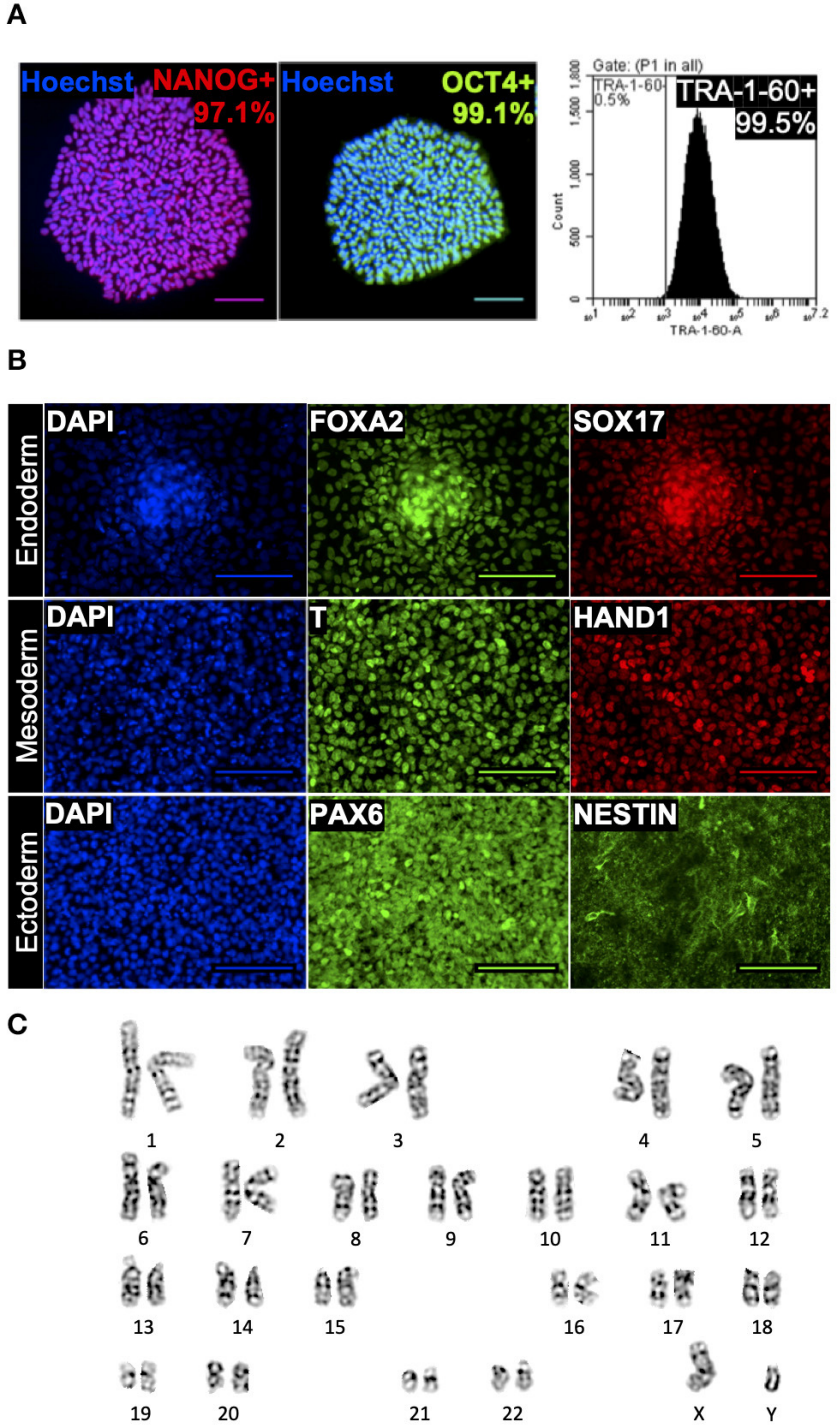
Immunocytochemistry of human induced pluripotent stem cells (hiPSCs) cryopreserved using the optimized dimethyl sulfoxide (DMSO)-free solution. Monochromatic images with pseudo-coloring matching the real color of respective fluorescent dye. **(A)** Quantitative fluorescent microscopy (counterstained with nuclear dye Hoechst 33342, blue) and forward vs. side scatter-gated flow cytometry of cryopreserved hiPSCs showing high expression of transcription factors NANOG (red), OCT4 (green), and pluripotency surface marker TRA-1-60. Scale bar: 100 μm. **(B)** Immunocytochemistry images showing trilineage differentiation of cryopreserved hiPSCs into three germ layers and expression of endodermal markers, FOXA2 and SOX17, mesodermal markers, T and HAND1, and ectodermal markers, PAX6 and NESTIN. Scale bar: 100 μm. **(C)** A representative image of normal male karyotype without numerical or structural chromosomal abnormality from the 16 metaphase cells available for analysis.

To evaluate the suitability of this optimized DMSO-free method for cell banking purposes, cells were characterized after freezing, thawing and one passage of post-thaw culture for three freeze-thaw cycles amplifying any phenotypic instability that could result from cryopreservation. The hiPSC aggregates cryopreserved using this method consistently yielded post-thaw culture with efficiency comparable to fresh cells passaged and better than cells cryopreserved in DMSO. Specifically, 24 h after plating at a split ratio of 1:6 (three independent experiments of six biological replicates), reattachment measured for cells cryopreserved using the optimized DMSO-free solution was similar to fresh cells (*p* > 0.05) and 52–95% (95% confidence interval) higher than cells cryopreserved using DMSO. On day 4 post-thaw, the cells cryopreserved using the optimized DMSO-free formulation exhibited high expression of NANOG, OCT4, and TRA-1-60 (Figure 3A) and demonstrated the ability to differentiate into cell types representative of all three germ layers (Figure 3B), illustrating that the cells retained their pluripotent phenotype and differentiation potential. In addition, samples of hiPSC aggregates were karyotyped after freezing, thawing and three passages of post-thaw culture for three freeze-thaw cycles amplifying any chromosomal instability that could result from cryopreservation. G-banding found a normal male karyotype with no clonal numerical or structural chromosomal abnormality in all 16 metaphase cells available for analysis (Figure 3C).

### Freezing Responses—Optimized vs. Non-optimized DMSO-Free Solution

As described in Figure 2, differences in CPA composition can have a profound effect on post-thaw cell survival, and higher levels of CPA did not always result in increased post-thaw cell survival. Two different DMSO-free solutions that appeared in the DE algorithm were tested and compared for their effect on the freezing responses of hiPSCs. Solution A was the optimized CPA solution containing level-2 sucrose, level-5 glycerol, level-1 isoleucine, and level-4 albumin. Solution B contained level-3 sucrose, level-4 glycerol, level-2 isoleucine, and level-5 albumin, which differed from the optimum by only one concentration level per CPA variable (i.e., 20 mM, 0.5% v./v., 7.5 mM, and 0.5%). Solution A resulted in post-thaw cell reattachment of ~100% when compared to fresh cells post-passage, whereas Solution B resulted in significantly lower post-thaw cell reattachment and cell losses of over 50% at 24 h after thawing (Table 3).

**Table 3:** Comparison of freezing responses in Solutions A, B, and C under optimal cooling rate of –1°C/min and ice nucleation temperature of –4°C^a^.

**Measurements**	**Solution A (optimized DMSO-free)**	**Solution B (non-optimized DMSO-free)**	**Solution C (optimized DMSO)**
Post-thaw cell reattachment rate, *n* = 18	104 ± 5.73%	48.7 ± 9.85%^*^	58.4 ± 6.58%^*^
Area fraction of ice in frozen solution, *n* = 5	76.0 ± 7.93%	80.3 ± 4.28%^n.s.^	68.6 ± 10.4%^n.s.^
Distance between adjacent ice crystals (μm), *n* = 20	2.16 ± 0.667	0.670 ± 0.400^*^	1.85 ± 0.952^n.s.^
Area fraction of intracellular ice in frozen cell aggregate, *n* ≥ 3	2.76 ± 1.58%	25.7 ± 23.9%^*^	16.6 ± 9.05%^*^
Proportion of cells that had intracellular ice, *n* = 12	0/12	6/12^*^	5/12^*^

a *Area fraction of ice, distance between adjacent ice crystals, area fraction of intracellular ice and proportion of cells with intracellular ice were quantified from Raman heat maps represented by Figure 4. DMSO: dimethyl sulfoxide. 95% confidence intervals calculated from samples of size shown for each metric. ANOVA with Bonferroni correction used to determine statistical significance compared to Solution A. ^n.s.^p > 0.05*;

* *p < 0.05*.

### Freezing Characteristics of Solutions A vs. B

Raman spectroscopy was used to characterize the freezing behavior of Solutions A and B. Effective cryopreservation of cells typically arises from the ability of CPA to control the freezing behavior of water, preventing the types of ice crystal formation that disrupt membrane integrity in suspended cells. When frozen alone without cells, Solution A formed ice crystals with a unique morphological characteristic not observed in Solution B: distinctly shown in both the lateral and axial Raman heat maps, each ice crystal in Solution A had a softened solid-liquid interface on one side and a regular, abrupt interface on the other side (Figures 4A,B). As a result, although the total amount of ice formation quantified by area fraction was similar between Solutions A and B, the distance between adjacent ice crystals was significantly greater in Solution A than Solution B (Table 3). As frozen cells are typically located in the space between adjacent ice crystals, the greater distance between ice could reduce the likelihood of intracellular ice formation and therefore cellular damage (Yu et al., 2017).

**Figure 4:**
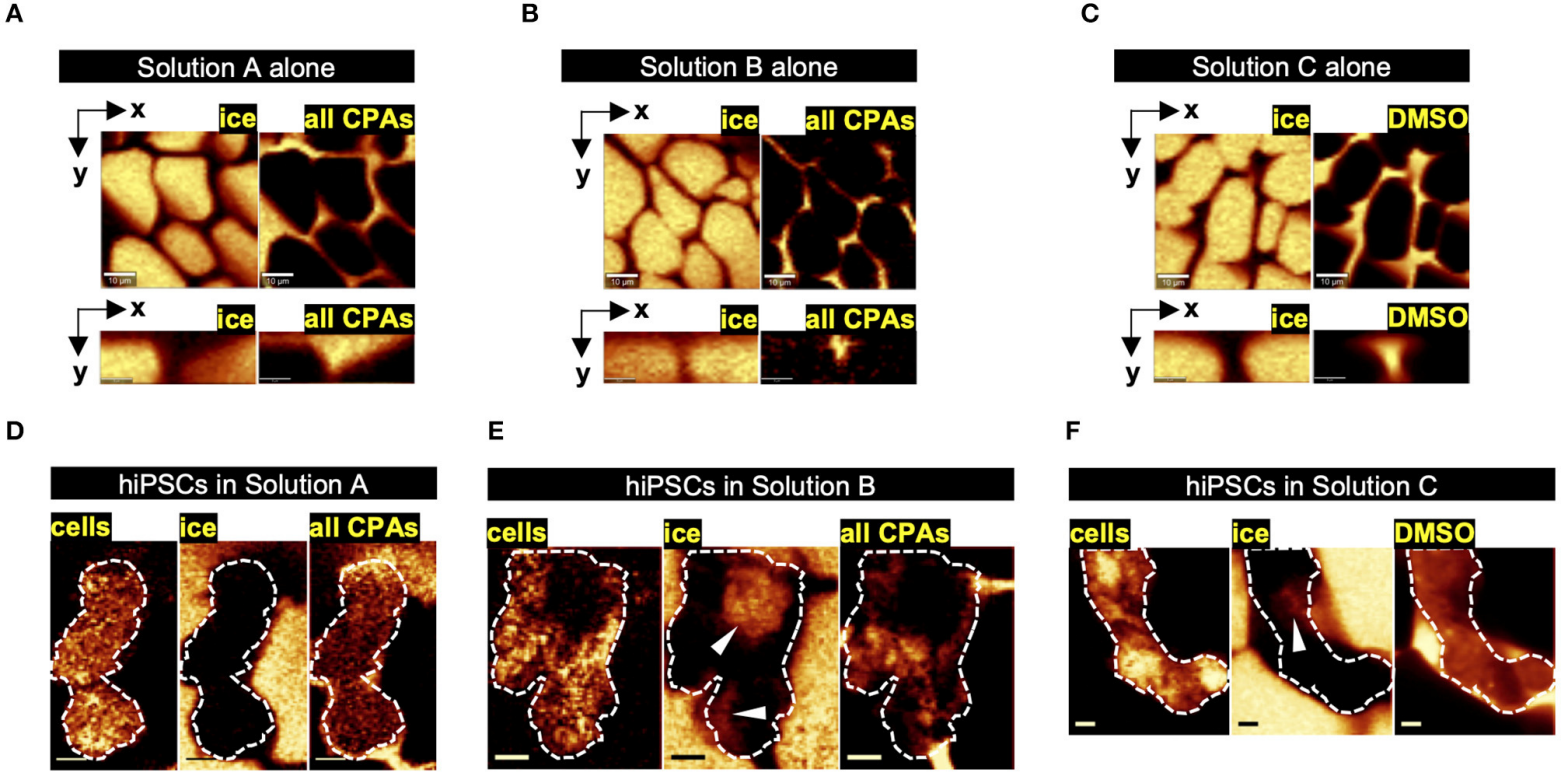
Raman heat maps representative of freezing responses in optimized dimethyl sulfoxide (DMSO)-free vs. non-optimized DMSO-free vs. optimized DMSO solutions. **(A–C)** Raman heat maps of ice crystals separated by non-frozen channels of the respective cryoprotective agent (CPA) solution at −50°C shown in lateral plane (scale bar: 10 μm) and axial plane (scale bar: 3 μm). **(D–F)** Raman heat maps of ice formation and CPA distribution in cell aggregate frozen in the respective solution at −50°C (scale bar: 5 μm), with the cell aggregate outlined (white dash) and intracellular ice marked (white arrowhead).

DSC was used to characterize the melting temperature, enthalpy of melting, and glass transition temperature of different CPA compositions (Table 4). Lower enthalpy of melting suggests lower amount of ice formed during freezing. There was no statistically significant difference in melting temperature, enthalpy of melting or glass transition temperature between Solutions A and B. Comparison of Solution A with and without two CPA components (isoleucine and albumin) showed no statistically significant difference in enthalpy of melting, indicating that albumin and isoleucine did not affect the amount of ice that formed during freezing. However, significant differences in melting temperature and enthalpy of melting were observed for Solution A with and without P188, indicating that the presence of P188 both depressed and suppressed ice formation during freezing. These DSC findings suggest that the difference in post-thaw cell survival between Solutions A and B likely resulted from interactions between components of the solution and different structures in the cell (membrane and proteins) rather than changes in the freezing behavior of water.

**Table 4:** Melting temperatures, enthalpy of melting, and glass transition temperatures for DMSO-free and DMSO CPA solutions measured by DSC^a^.

**CPA solution**	***T_**m1**_*(°C)**	***T_**m2**_*(°C)**	***ΔH_**m**_* (J/g)**	***T_**g**_* (°C)**
A (optimized DMSO-free)	−6.727 ± 0.712	−2.047 ± 0.0940	205.87 ± 21.49	−89.150 ± 1.631
A minus albumin	−6.770 ± 0.433	−1.830 ± 0.293	219.90 ± 13.12	−89.557 ± 5.594
A minus isoleucine	−7.027 ± 0.296	−1.913 ± 0.117	212.20 ± 4.390	−89.807 ± 2.414
A minus P188	−6.257 ± 0.0759*	−1.697 ± 0.2748*	230.47 ± 15.68*	−91.693 ± 0.737
B (non-optimized DMSO-free)	−6.623 ± 0.826	−1.903 ± 0.225	210.97 ± 27.53	−88.637 ± 4.875
C (optimized DMSO)	−8.013 ± 0.263*	−2.527 ± 0.123*	214.08 ± 9.392	−118.45 ± 0.723*
P188 alone	−2.903 ± 0.972	0.537 ± 0.857	254.07 ± 63.37	−69.610 ± 2.218

a DMSO, dimethyl sulfoxide; CPA, cryoprotective agent; DSC, differential scanning calorimetry; T_m1_, melting temperature defined as the onset of melting; T_m2_, melting temperature defined as the peak of melting; ΔH_m_, enthalpy of melting; T_g_, glass transition temperature; A, Solution A; B, Solution B; C, Solution C; P188, poloxamer 188. Measurements shown as 95% confidence intervals. Asterisk (*) indicates statistical significance using a two-sample t-test (p < 0.05) compared to Solution A.

### Cell Response to Freezing in Solutions A vs. B

Raman spectroscopy was also used to characterize the freezing response of hiPSC aggregates in the two solutions of interest. Freezing of hiPSCs in Solution A resulted in the formation of little to no intracellular ice, whereas freezing of hiPSCs in Solution B resulted in significantly more intracellular ice (Table 3). None of the cells (0/12 cells) frozen in Solution A exhibited intracellular ice formation, whereas 6 out of 12 cells contained intracellular ice when frozen in Solution B. As represented by Figure 4E, the ice crystals found inside the cell aggregates frozen in Solution B were near or in direct contact with extracellular ice, suggesting that the intracellular ice was nucleated by extracellular ice on the other side of the plasma membrane. The fraction of cells exhibiting intracellular ice was consistent with the post-thaw cell reattachment rates of the two solutions. The Raman heat maps also permitted quantification of CPAs partitioning across the cell membrane, an indicator of membrane integrity. As expected, sucrose was non-penetrating and located outside of the hiPSCs, and glycerol penetrated the plasma membrane of the cells in Solution A, indicating normal membrane permeability (Figure 4D). However, higher concentration of sucrose and glycerol was found inside the cells in Solution B, which is consistent with a loss of membrane integrity (Figure 4E).

### Cell Sensitivity to Undercooling in Solutions A vs. B

**Figure 5:**
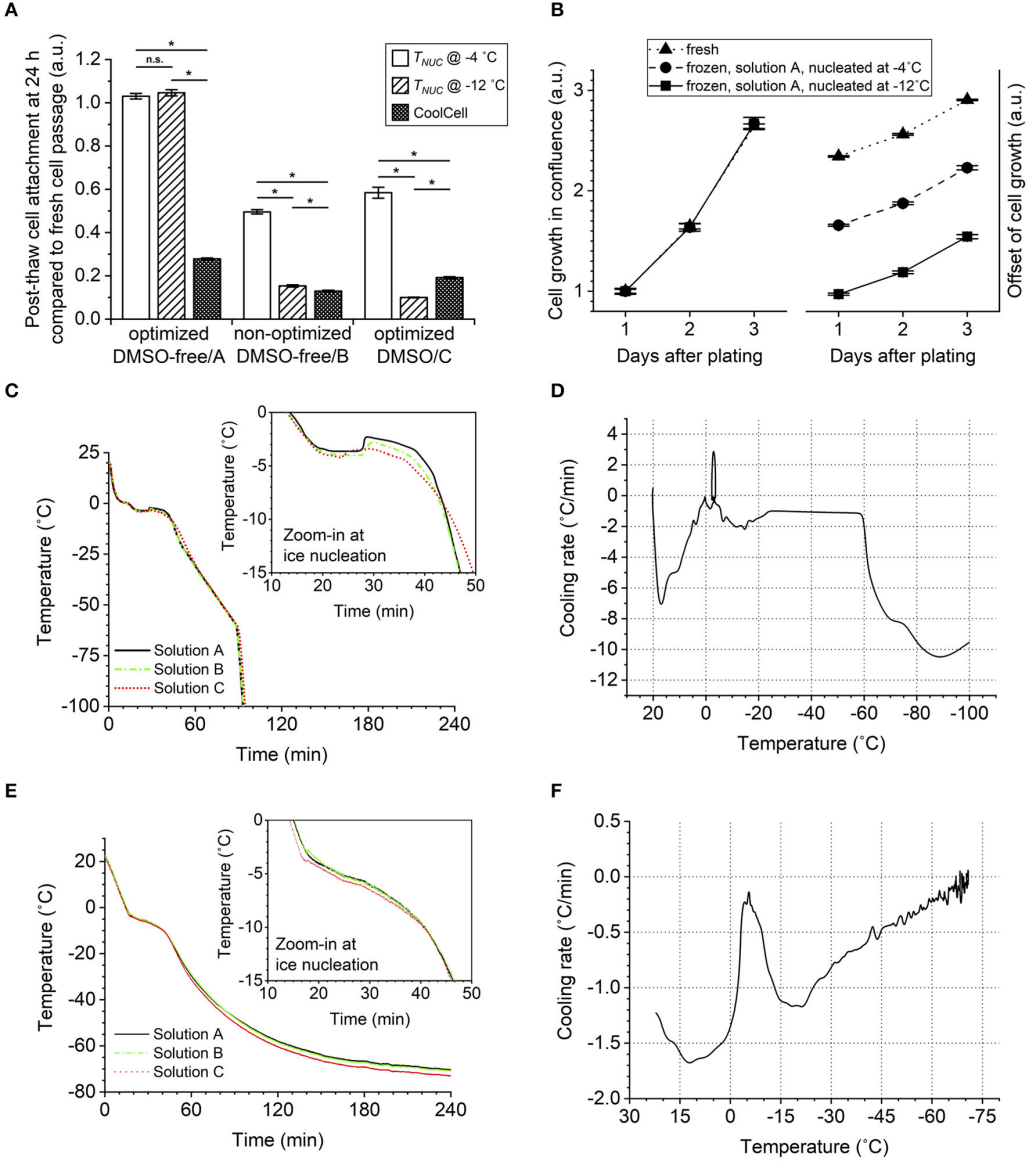
Effect of ice nucleation temperature (undercooling) on post-thaw cell survival in conditions of different cryoprotective agent (CPA) solutions. Error bars of standard error. **(A)** Cell reattachment measured by calcein AM staining 24 h after thawing at 1:6 split ratio, showing greater cell sensitivity to the freezing condition when using the non-optimized dimethyl sulfoxide (DMSO)-free or optimized DMSO solution than the optimized DMSO-free solution. ANOVA with Bonferroni correction performed for comparison. ^n.s.^*p* > 0.05; *p < 0.05. Sample size of 18 (three independent experiments of six) biological replicates. **(B)** Cell growth in terms of culture confluence monitored for 3 days after thawing or passaging at 1:6 split ratio, showing normal growth rate of cryopreserved cells not affected by the ice nucleation temperature (*T*_*NUC*_). Sample size of six biological replicates. **(C)** Sample internal temperature recorded over the course of controlled-rate freezing with ice nucleation induced manually at −4°C. **(D)** First derivative of **(C)** plotted against the corresponding sample internal temperature. **(E)** Sample internal temperature recorded over the course of passive freezing in CoolCell with spontaneous ice nucleation. **(F)** First derivative of **(E)** plotted against the corresponding sample internal temperature.

In a previous study (Li et al., 2018), we demonstrated that hiPSCs in DMSO were sensitive to undercooling (the difference between the melting temperature and the temperature at which ice forms or ice nucleation temperature). Based on the DSC measurements of peak of melting (*T*_*m*2_) shown in Table 4, the extent of undercooling was similar (*p* > 0.05) between Solutions A and B when ice nucleation was induced at the same temperature in the respective samples. The extent of undercooling was ~2°C when ice nucleation was induced at −4°C and increased to 10°C when ice nucleation was induced at −12°C. The sensitivity of hiPSC aggregates frozen in the two DMSO-free formulations of interest to undercooling was compared. As shown in Figure 5A, decreasing ice nucleation temperature from −4 to −12°C did not affect the post-thaw reattachment of cells cryopreserved in Solution A. In contrast, high sensitivity to undercooling was observed when the formulation was shifted away from the optimum. Cells cryopreserved in Solution B showed significantly lower post-thaw cell survival with decreasing ice nucleation temperature. For hiPSCs cryopreserved in Solution A, the cell growth rates measured over a 3-days period after seeding were very similar among fresh cells and frozen cells nucleated at −4 or −12°C (Figure 5B). This outcome indicated that both the post-thaw reattachment and the growth rate of the cells cryopreserved in Solution A were similar for the different ice nucleation temperatures tested.

### Freezing Responses—Optimized DMSO-Free vs. DMSO Solution

The next phase of this investigation involved a comparison of Solution A, the optimized DMSO-free composition, and Solution C, a 7.5% DMSO solution commonly used to cryopreserve hiPSCs (Martín-Ibáñez et al., 2012).

### Freezing Characteristics of Solutions A vs. C

Raman spectroscopy was used to characterize the freezing behavior of Solutions A and C. Compared to the unique morphology of ice that formed in Solution A, the ice crystals formed in Solution C had typical, abrupt solid-liquid interfaces (Figure 4C). The total amount of ice measured in a given field of view for each solution (Figures 4A,C) was not different (*p* > 0.05, Table 3). In addition, the distance between adjacent ice crystals was similar between the two solutions *(p* > 0.05, Table 3). DSC was used to compare the thermophysical behavior of Solutions A and C. As shown in Table 4, Solution C had a statistically significant lower melting temperature than Solution A (with or without P188). However, the total amount of ice measured by the enthalpy of melting was not different (*p* > 0.05) between Solutions A and C, which was consistent with Raman measurements described above. Despite its higher melting temperature, Solution A resulted in significantly better post-thaw cell survival when it was used to cryopreserve the hiPSC aggregates, whereas the lower melting temperature of Solution C, a typically desirable property of CPAs, was not sufficient to result in high levels of post-thaw cell survival. Solution C also exhibited glass transition temperature significantly lower than Solution A.

### Cell Response to Freezing in Solutions A vs. C

Low-temperature Raman spectroscopy was used to characterize the freezing responses of hiPSC aggregates in the two solutions. While Solution A resulted in little to no intracellular ice, Solution C resulted in significantly more intracellular ice (Table 3). While none of the cells (0/12) frozen in Solution A contained intracellular ice, 5 out of 12 cells frozen in Solution C were found with intracellular ice crystals. This outcome was consistent with the percent cell loss measured by post-thaw cell reattachment, suggesting that similar to Solution B, intracellular ice formation likely damaged the cells during freezing when Solution C was used and resulted in cell death or impaired cell adhesion after thawing.

As shown in Figure 4F, similar to cells frozen in Solution A, those frozen in Solution C showed normal permeability of the plasma membrane (i.e., normal partitioning of DMSO-free CPAs or DMSO inside and outside the cell aggregates), suggesting intact membrane integrity. However, Raman heat maps rendered from signals of proteins and lipids showed high variance in the distribution of protein and lipid content in the cells frozen in Solution C. A considerable amount of pixels were saturated in these Raman signals of interest, suggesting aggregation of cellular materials, such as organelles and cytoskeleton, while other parts of the cells contained dark regions with very low Raman signals, suggesting loss of cellular integrity. This outcome was not observed in the cells frozen in Solution A.

### Cell Sensitivity to Undercooling in Solutions A vs. C

Cell sensitivity to undercooling was also compared between the two solutions. When ice nucleation was induced at −12°C, both solutions were subject to 8°C greater undercooling than when ice nucleation was induced at −4°C. Based on measurements of peak of melting shown in Table 4, for a given ice nucleation temperature, the extent of undercooling was always 0.48°C greater in Solution A than in Solution C (*p* < 0.05). In spite of this slight advantage of Solution C, hiPSC aggregates in Solution C were sensitive to undercooling, whereas those in Solution A were not. Consistent with the results from a recent study that used DMSO to cryopreserve a different cell line of hiPSCs (Li et al., 2018), post-thaw reattachment of the cells cryopreserved in Solution C dropped significantly with lower ice nucleation temperature (Figure 5A). When ice nucleation occurred at −12°C, post-thaw cell reattachment was 9.53–10.4% (95% confidence interval) in Solution C, while no cell loss was observed in Solution A for the same ice nucleation temperature.

It was also noteworthy that passive freezing of hiPSC aggregates using insulated containers, such as a CoolCell in a −80°C mechanical freezer, resulted in significant cell loss regardless of CPA composition (Figure 5A). The increase in temperature associated with the release of latent heat (Figure 5E) suggests that nucleation occurred approximately between −3 and −4°C for all solutions tested. Therefore, there was no evidence of excessive undercooling that would lead to cell loss in the CoolCell. The temperature-time profiles (Figures 5C,E) and the corresponding cooling rate experienced by the samples (Figures 5D,F) indicate that lower cooling rates were observed using the CoolCell than using the controlled-rate freezer, which can explain the cell loss.

## Discussion

In this study, the DE algorithm produced an optimal DMSO-free formulation for the cryopreservation of hiPSCs under a small number of experiments. Traditional approaches, such as empirical trial-and-error and high-throughput screening would take up to 1,296 experiments to locate an optimum in this multi-dimensional parameter space; this algorithm reduced the amount of experimentation to only eight experiments. Alternative methods of optimization are design of experiment (DOE) and the Taguchi method, which is a variant of DOE. Both DOE and Taguchi methods assume that the topology of the parameter space that is being studied is unimodal and that there are no interactions between parameters. Both of these assumptions are not true, as this study has demonstrated that the topology is not unimodal, and our statistical model (Pi et al., 2019b) has shown that that there are synergistic effects between the CPA molecules tested, making these alternative approaches unsuitable. Emergent population staying constant for two generations was defined as the criterion of convergence for the DE algorithm. This criterion has been validated in our recent study (Pi et al., 2019a) to ensure ≥95% accuracy of predicting the global optimum.

Compared to our previous DE algorithm studies (Pollock et al., 2017; Pi et al., 2019a), this study optimized four variable components instead of the former three variables, which resulted in six times as many possible vectors in the parameter space than before. However, the population size of each generation (i.e., 10) was smaller than what had been commonly used before (i.e., 13–27). Interestingly, the convergence speed (i.e., seven iterations) was well-maintained in spite of the higher-dimensional parameter space and smaller experiments. It is also noteworthy that this study used the attachment of multicellular aggregates, a more complex biological system than previously reported and a functional metric. All of these further provide evidence for the stability of this DE algorithm and demonstrate its promising ability to achieve optimization with a small number of cells. With the capability of handling designs and data of greater complexity but no limit to the number of parameters or the type of application beyond cryopreservation, the DE algorithm could serve as a powerful tool to accelerate the otherwise resource-intensive process of optimization and encourage the beneficial practice of optimizing translational stem cell technologies in general.

A variety of methods for preserving hiPSCs have been developed (see review article; Li and Ma, 2012). These methods can be divided into two different categories: (1) vitrification and (2) slow freezing. DMSO has been a universal component in these cryopreservation solutions. ROCK inhibitor is commonly used as an additive to suppress post-thaw apoptosis (Hunt, 2011; Katkov et al., 2011; Beier et al., 2013; Imaizumi et al., 2014). In contrast, this investigation describes a DMSO-free method that freezes hiPSCs in aggregates and avoids the use of ROCK inhibitor.

The combination of previous studies (Pollock et al., 2016; Pi et al., 2019b) and new findings from this investigation provides insights into how the DMSO-free CPAs act to protect the hiPSCs from freezing damage. Mechanisms of cryoprotection for DMSO-free CPAs differ from that of DMSO, which is an organic solvent. Both sucrose and glycerol influence the hydrogen bonding with water (Dashnau et al., 2005, 2006; Yu et al., 2018) and interact with each other (Pi et al., 2018) to likely form natural deep eutectic systems (NADES) (Castro et al., 2018). These molecular interactions can modify the freezing behavior of water around cells, specifically changing ice crystal morphology to reduce cellular damage from ice.

The CPAs used also interact with the biological structures in cells. Low-temperature Raman spectroscopy has demonstrated that sucrose interacts with the plasma membrane (Yu et al., 2018). While cellular dehydration plays a major role in preventing intracellular ice, glycerol also inhibits intracellular ice by forming strengthened hydrogen bonds with the remaining water content in the cytoplasm (Dashnau et al., 2006; Towey and Dougan, 2012). Isoleucine was found to be cryoprotective at a low concentration, while higher concentrations resulted in lower post-thaw cell survival. While DSC results of the thermophysical properties of CPAs in this study showed that isoleucine did not significantly contribute to the ice inhibition effects of the CPA cocktail overall, DE algorithm results showed that post-thaw cell survival could be increased significantly (e.g., from 48.3 to 100%) by 7.5 mM isoleucine. This result is likely a “sweet spot” between the stabilizing (Brennan et al., 2003; Bozorgmehr and Monhemi, 2015) and destabilizing (Taneja and Ahmad, 1994) effects of a hydrophobic amino acid on protein molecules in the cells. While albumin may not have a significant effect on ice formation as shown by DSC, it can act as a scavenger of free radicals alleviating oxidative stress and a carrier of waste produced from any cell damage and stress in freezing and thawing (Boldt, 2010). Among the thermophysical properties of a CPA solution, melting temperature indicates the ability of the solution to depress ice crystallization, enthalpy of melting correlates to the amount of ice formed, and glass transition temperature indicates the extent of partial vitrification at low temperatures.

Most notably, we were able to use Raman spectroscopy and DSC to characterize a novel additive for hiPSC cryopreservation, P188. P188 was found here to not only inhibit ice formation significantly but also soften the solid-liquid interface of ice and increase the distance between adjacent ice crystals. These changes in ice crystal formation outside the cells can in turn suppress ice formation inside the cells (Yu et al., 2017). It is important to understand that these cryoprotective effects of the DMSO-free CPA cocktail could be capitalized only with the optimized composition. Deviation from the optimum could result in significantly less desirable outcomes in hiPSC cryopreservation as shown in the results. When a non-optimized DMSO-free solution is used to cryopreserve hiPSC aggregates, formation of intracellular ice is changed, and cells are significantly less likely to survive post-thaw. One potential explanation for this outcome is that the optimal DMSO-free CPA composition results in enhanced stabilization of the cell membrane and thereby confers greater resistance to intracellular ice formation. Further study will be needed to validate the cryoprotective effects of this optimal DMSO-free CPA cocktail using a variety of hiPSC cell lines.

Our earlier study found that hiPSC aggregates were more sensitive to undercooling than single cells frozen in DMSO (Li et al., 2018). The sensitivity to undercooling has been observed in a variety of biological systems. As described in a review article (Morris and Acton, 2013), controlled ice nucleation of samples or directional solidification, which eliminates undercooling, has been shown to improve post-thaw cell recovery. The outcome of this investigation expands our understanding of this issue. Specifically, cell aggregates frozen in DMSO and a suboptimal formulation of a DMSO-free solution exhibited sensitivity to undercooling. This behavior restricts the modalities of freezing suitable for hiPSCs in these solutions. However, hiPSC aggregates cryopreserved using an optimized DMSO-free solution exhibited far less sensitivity to undercooling, and ice nucleation temperatures of as low as −12°C do not result in cell losses post-thaw. In contrast to DMSO, the optimal DMSO-free solution provides greater flexibility in freezing and can accommodate greater variability in execution of the freezing protocol. In design of a controlled-rate freezing protocol, it is important that the ice nucleation (or seeding) temperature is lower than the peak temperature of melting for the CPA solution used to ensure that ice crystallization is feasible and that the frozen state can be maintained in the subsequent slow freezing steps. While ice nucleation at −4°C optimized in our earlier study (Li et al., 2018) demonstrated effective cryopreservation using the optimized DMSO-free solution in this study, ice nucleation temperatures below −2.2°C and above −4°C may be alternative ice nucleation temperatures to be used with this method in future studies. In addition, the use of passive freezing devices resulted in a significant drop in post-thaw cell survival when compared to controlled-rate freezing. This observation is consistent with the sensitivity of hiPSCs to cooling rate. The outcome of this investigation enables not only improved cryopreservation of hiPSCs but also insight into the sensitivity of multicellular systems to freezing and strategies to overcome those sensitivities.

## Data Availability Statement

All datasets generated for this study are included in the article/Supplementary Material.

## Author Contributions

RL, JD, and AH: conception and design. RL and KH: acquisition, analysis, and interpretation of data. RL, KH, and AH: draft manuscript writing. RL, KH, JD, and AH: manuscript revision and final approval of manuscript.

### Conflict of Interest

The authors declare that the research was conducted in the absence of any commercial or financial relationships that could be construed as a potential conflict of interest.

## Supplementary Material

Supplementary Figure 1 Cooling profiles of optimized controlled-rate freezing at −1°C/min with ice nucleation manually **(A)** or automatically **(B)** induced at −4°C. Stepwise protocol of each method shown under graph. T_*NUC*_, ice nucleation temperature; *B*, cooling rate. **(A)** Ice nucleation induced by manually spraying liquid nitrogen (LN2) onto cryogenic vials. Sample internal temperature showing minor release of latent heat at step 3 indicating good inhibition of ice formation by the cryoprotective agents (CPAs). **(B)** Ice nucleation induced by a programmed dip. Sample internal temperature showing minor latent enthalpy dissipated by the low temperature of the freezer chamber at step 2 indicating good inhibition of ice formation by the CPAs.
