# Supplementary figures and images for: Cryopreservation of Human iPS Cell Aggregates in a DMSO-Free Solution—An Optimization and Comparative Study
Source: Front Bioeng Biotechnol. 2020 Jan 22;8:1. doi: 10.3389/fbioe.2020.00001 (PMC6987262; doi:10.3389/fbioe.2020.00001)

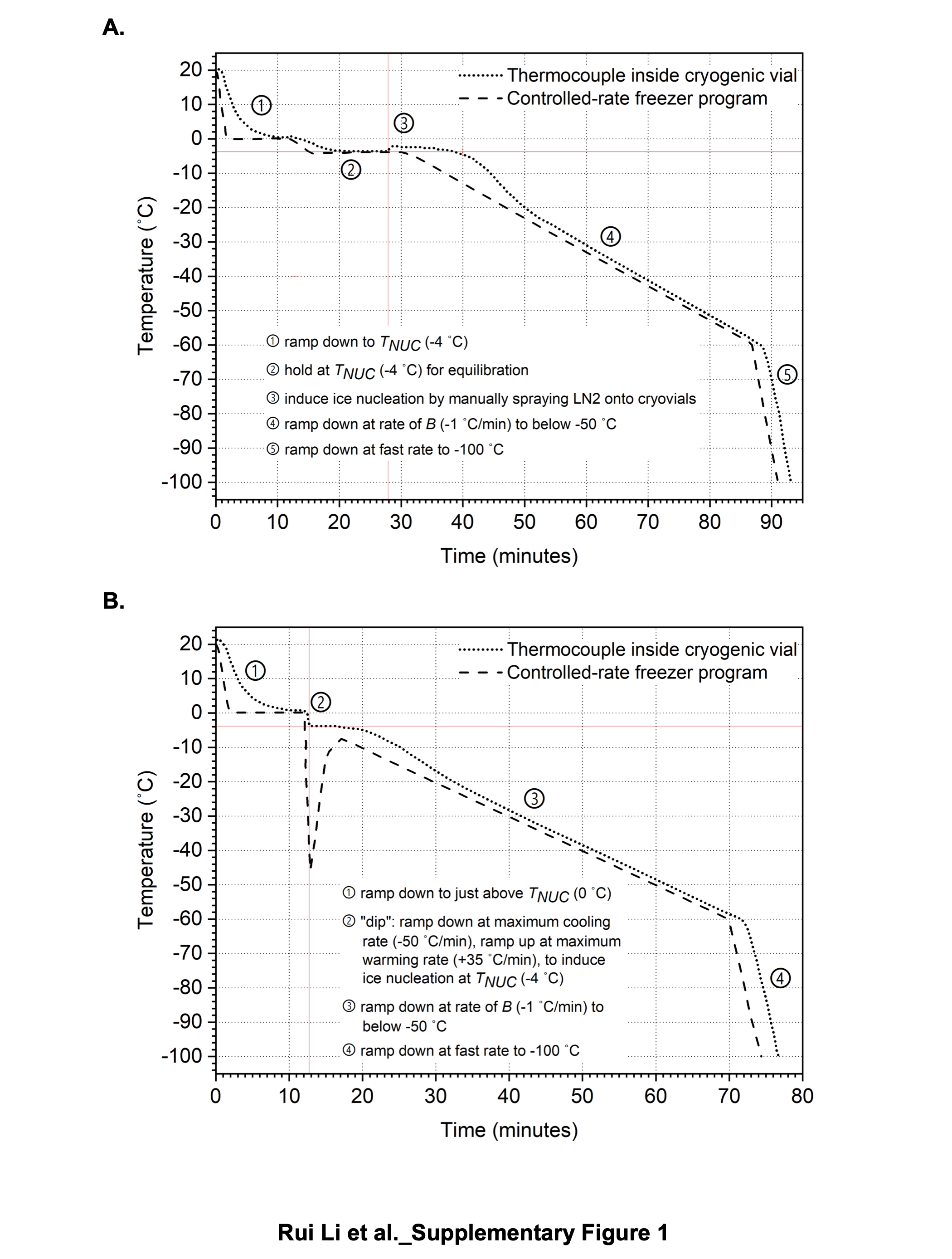

Supplement: Supplementary Figure 1 — Cooling profiles of optimized controlled-rate freezing at −1°C/min with ice nucleation manually (A) or automatically (B) induced at −4°C. Stepwise protocol of each method shown under graph. TNUC, ice nucleation temperature; B, cooling rate. (A) Ice nucleation induced by manually spraying liquid nitrogen (LN2) onto cryogenic vials. Sample internal temperature showing minor release of latent heat at step 3 indicating good inhibition of ice formation by the cryoprotective agents (CPAs). (B) Ice nucleation induced by a programmed dip. Sample internal temperature showing minor latent enthalpy dissipated by the low temperature of the freezer chamber at step 2 indicating good inhibition of ice formation by the CPAs. [file Image_1.TIF]
